# Editorial: Grief After Suicide: A Health Perspective on Needs, Effective Help, and Personal Growth

**DOI:** 10.3389/fpsyg.2020.614405

**Published:** 2020-11-17

**Authors:** Karl Andriessen, Karolina Krysinska, Dolores Angela Castelli Dransart

**Affiliations:** ^1^School of Population and Global Health, The University of Melbourne, Parkville, VIC, Australia; ^2^School of Social Work Fribourg, University of Applied Sciences and Arts of Western Switzerland, Fribourg, Switzerland

**Keywords:** bereavement, grief, grief after suicide, helpseeking, postvention, suicide, personal growth

Experiencing the suicide of a close person often signifies a major disruptive stressor, exacerbating the risks of social, physical, and mental health problems, and suicidal behavior in the bereaved individuals (Pitman et al., 2014). Common grief reactions after any death include feelings of sadness, longing, guilt, and anger. Compared to other forms of bereavement, people bereaved by suicide may experience more shock or trauma related to the unexpected or violent nature of the death, and more feelings of abandonment, rejection, and shame (Jordan and McIntosh, 2011a). They may struggle more with meaning-making and “why” -questions, and experience less social support (Feigelman et al., 2009; Castelli Dransart, 2013).

Compared with the general population, people bereaved by suicide have a higher risk of suicidal behavior, and mental health problems such as depression, anxiety, posttraumatic stress disorder (PTSD), and substance abuse (Erlangsen et al., 2017). Suicide bereavement also represents a risk factor for complicated grief (Bellini et al., 2018). Those bereaved who have a personal or family history of mental health and/or suicidal behavior appear to be more vulnerable to the negative psychosocial outcomes (Andriessen et al., 2016; Pitman et al., 2016). Despite these challenges, people bereaved by suicide can also experience personal and posttraumatic growth (Castelli Dransart, 2016; Genest et al., 2017; Levi-Belz et al., 2020). Research has shown that ~1 in 5 people may experience a suicide during their lifetime highlighting the public and mental health importance of loss by suicide (Andriessen et al., 2017c).

This Research Topic aimed to broaden our understanding of grief after suicide, with regards to the needs of bereaved individuals and communities, and how to best help the bereaved, within a health psychology context. As such, the Research Topic expands the focus of previous work in this field (Jordan and McIntosh, 2011b; Andriessen et al., 2017b). The 16 published studies fall into three broad categories: (a) the experience of suicide bereavement (8 studies), (b) the impact of a death by suicide on professionals (3 studies), and (c) interventions (5 studies).

## Experiences of Suicide Bereavement

Regarding the experience of suicide bereavement, Feigelman and Cerel (USA) conducted a survey investigating feelings of blameworthiness in bereaved parents. Blameworthiness related to participants' perception of what they may have done (or not done) that could have contributed to their child's death, including suicide. The study found that feelings of blameworthiness strongly correlated with grief difficulties and mental health problems, such as complicated grief, PTSD, and depression.

Levi-Belz and Gilo (Israel) explored the moderating role of self-forgiveness regarding emotional distress in people bereaved by suicide. The study found that accepting one's mistakes and fostering positive emotions, thoughts, and behaviors toward oneself can be a protective factor against depression and suicidality in people bereaved by suicide. The study findings indicated the possible efficacy of forgiveness-based interventions in this population.

Two qualitative studies focused on suicide bereavement in the context of older adults. Hybholt et al. (Denmark) studied older adults' conduct of everyday life during the first 5 years after the death. The study identified age-related issues in the participants' bereavement process. Life was no longer as expected (“the broken notion of late-life living”) and participants perceived limited possibilities and time to restore their life project. Age-related factors affected their possibilities to adjust to their new life conditions.

Michaud-Dumont et al. (Canada) explored the experience of family members bereaved by the suicide of a close elderly relative. This small pilot study revealed how participants searched explanations for the suicide. Despite the perceived inevitability of death in older age, participants reported that the suicide was shocking and unexpected, and triggered strong emotions, such as anger and guilt, as well as family conflicts. The pilot study elucidated important methodological issues for future studies, for example, regarding recruitment.

Two studies looked at suicidal behavior in people bereaved by suicide. Westerlund et al. (Sweden) conducted a survey with women bereaved by suicide. It appeared that the self-reported rates of suicidal thoughts, plans, and attempts were considerably higher in this group than the rates reported in the general population. Having lost a child (as compared to another family member), shame, and experiencing family avoidance increased the risk of suicidality, leading the authors to conclude that postvention activities should target these factors.

Based on the Integrated Motivational-Volitional Model of Suicide (O'Connor and Kirtley, 2018), del Carpio et al. (Scotland/UK), examined bereavement by suicide or other death as a longitudinal predictor of self-harm in adolescents. The study reported on the prevalence of suicide bereavement and self-harm in adolescents, as well as various risk factors. Neither bereavement by suicide nor by non-suicide did predict self-harm in the bereaved adolescents.

Pitman et al. (UK) conducted a mixed methods study on self-reported use of alcohol and unprescribed drugs following loss by suicide or other sudden deaths in young people. There was no increase in alcohol or drug use in more than half of the bereaved young people. Nonetheless, young people bereaved by suicide or non-suicide unnatural deaths were more likely to report higher substance use than those bereaved by sudden natural causes.

De Leo et al. (Italy) systematically reviewed the literature on how a death can be communicated. The review found that death notification is a complex and stressful experience both for those who provide the information and for the bereaved individuals who receive the news. This process requires high-quality training and flexible protocols tailored to particular sets of circumstances.

## Impact of Suicide in Health Professionals

Three studies focused on the impact of a suicide death on professionals in mental health and health settings. In a qualitative study, Nelson et al. (UK) explored the perspectives of ambulance staff on attending to deaths by suicide. The study revealed that responding to suicide can have professional and personal impact on the ambulance staff, including job-related strain and long-term traumatic memories. Training regarding how to respond to people bereaved by suicide and debrief opportunities were rare, pointing at a need for training and support for ambulance staff.

Rothes et al. (Portugal) interviewed prehospital health professionals regarding their experiences with emergency patients dying by suicide. Participants reported that the suicide of a patient had intense negative impacts, such as intrusive thoughts and images, and doubts about professional competence and liability. However, participants also spoke of potential positive effects, such as professional growth and increased awareness. The study stressed the importance of training for prehospital health staff.

Leaune et al. (France) presented a protocol for a mixed methods collaborative and participatory action research, the “SUPPORT-S” study. This study will evaluate the implementation and effectiveness of the SUPPORT postvention program, which provides comprehensive support to mental health and social work professionals impacted by exposure to patient suicide.

## Interventions

Five studies dealt with postvention interventions in different settings. Andriessen et al. (Australia) systematically reviewed the peer reviewed and gray literature, and presented an overview of recent models and guidelines for suicide postvention services, as well as components that may contribute to their effectiveness. The review recommended adopting a public health framework to tailor support to bereaved individuals according to the impact of suicide on their lives. Such support may range from information and awareness raising to specialized psychotherapy.

Berardelli et al. (Italy) presented a weekly group program for people bereaved by suicide, facilitated by trained psychologists. The program aimed to provide support, normalize grief reactions, and integrate the loss, and was well received by the participants. The psychoeducational approach allowed participants to interact with each other, helped them resume the course of life and place the suicide of a close person in a broader perspective.

Hagström (Sweden) qualitatively investigated how a theater play might counteract the stigmatized trauma of suicide bereavement. The study concluded that research-based theater can resonate well with the experiences of the bereaved individuals and is a promising cost-effective means of creating new meanings around suicide, both for individuals bereaved by suicide and the broader cultural context from which stigma originates.

Geležėlytė et al. (Lithuania) in a mixed methods study explored factors contributing to seeking professional psychological help by people bereaved by suicide. The findings indicated that those bereaved who experienced more stigmatization and guilt might contact professionals more often. Attitudes toward mental health specialists was the strongest predictor of help-seeking in participants. Conversely, gaps in the health care system was identified as a main barrier.

Jordan (USA) shared “lessons learned” over 40 years of his work as a grief therapist with people bereaved by suicide. This insightful paper identified a series of psychotherapeutic tasks regarding the psychological integration of the loss, such as containment of the trauma and restoration of a sense of psychological safety, psychoeducation about suicide, trauma and grief, repairing the continuing bond with the deceased, and rebuilding an assumptive world that has been shattered by the suicide. The paper concluded with clinical implications for the work with people bereaved by suicide.

## Conclusions

The published papers have clearly advanced the knowledge and insights in postvention. They addressed many priority topics identified by those working in this field, such as theory-driven and longitudinal research, suicide bereavement in the contexts of older adults and helping professionals, suicidal behavior in suicide bereaved individuals, and evaluation of postvention programs (Andriessen et al., 2017a). The studies also highlighted important targets for interventions, such as self-blame and self-forgiveness, alcohol and substance use, suicidality in the bereaved, and facilitation of personal growth. Adopting a public health approach would allow tailoring interventions to the needs and the level of impact of the grief in those bereaved.

This Research Topic constitutes the largest open access peer-reviewed collection of studies regarding grief after suicide and is a testament to the substantial progress that has been made over the last years. We are grateful to the authors for submitting their manuscripts and for sharing their expertise. We are convinced that the published studies will be highly useful for clinicians, peer supporters, researchers and anyone involved in this field. These studies will inform further research and evidence-based training and interventions in postvention.

## Author Contributions

KA, KK, and DC have written the editorial together and have agreed on the final version. All authors contributed to the article and approved the submitted version.

## Conflict of Interest

The authors declare that the research was conducted in the absence of any commercial or financial relationships that could be construed as a potential conflict of interest.
